# Long-term micturition problems of asymptomatic postpartum urinary retention: a prospective case–control study

**DOI:** 10.1007/s00192-017-3457-6

**Published:** 2017-09-04

**Authors:** Femke E. M. Mulder, Robert A. Hakvoort, Jan-Peter de Bruin, Erica W. Janszen, Joris A. M. van der Post, Jan-Paul W. R. Roovers

**Affiliations:** 1 Department of Obstetrics and Gynaecology, Academic Medical Centre, Meibergdreef 9—room H4.240, 1105 AZ Amsterdam, The Netherlands; 2 Department of Obstetrics and Gynaecology, Martini Hospital, Groningen, The Netherlands; 3 Department of Obstetrics and Gynaecology, Jeroen Bosch Ziekenhuis, ‘s Hertogenbosch, The Netherlands; 4 Department of Obstetrics and Gynaecology, Onze Lieve Vrouwe Gasthuis, Amsterdam, The Netherlands

**Keywords:** Postpartum urinary retention, Covert postpartum urinary retention, Micturition symptoms, Incomplete bladder emptying, Post-void residual volume, Puerperium

## Abstract

**Introduction and hypothesis:**

Covert (asymptomatic) postpartum urinary retention (PUR) is defined as post-void residual volume (PVRV) ≥150 mL. Although often supposed to be a common and harmless phenomenon, no data are available on the potential long-term micturition problems of increased PVRV after vaginal delivery.

**Methods:**

After the first spontaneous void post-vaginal delivery, PVRV was measured using a portable scanning device. Micturition symptoms were compared using validated questionnaires between women with PVRV < 150 mL and those with PVRV ≥150 mL until 1 year after delivery. Women with PVRV ≥ 150 mL were followed until complete bladder emptying was achieved.

**Results:**

Data of 105 patients with PVRV < 150 mL and 119 with PVRV ≥ 150 mL were available for analysis. 75% of all patients included had PVRV ≥ 250 mL. More primiparous patients had PVRV ≥ 150 mL (*p* < 0.02). 92% of women with PVRV ≥ 150 mL after delivery were able to adequately empty their bladder within 4 days. One year after delivery, no statistically significant differences were found.

**Conclusions:**

Covert PUR according to the definition of PVRV ≥ 150 mL, is a common and transient phenomenon that does not result in more lower urinary tract symptoms 1 year after delivery. Although the current definition is not useful in identifying postpartum women with a pathological condition, we suggest that the definition of covert PUR should be change to: “PVRV≥500 mL after the first spontaneous void after (vaginal) delivery.” This cut-off value is the value at which some women do need more time to normalise emptying of the bladder. The exact clinical implications of covert PUR need to be further studied in this subcategory of women.

## Introduction

Postpartum urinary retention (PUR) is a frequently occurring condition in women [1–3]. Two types of PUR can be distinguished; overt (symptomatic) PUR, i.e., the inability to void spontaneously after delivery, and covert (asymptomatic) PUR, defined as abnormal post-void residual volumes (PVRVs) after micturition. By definition, the last form remains unrecognised when screening for residual volumes is not part of standard postpartum care. Therefore, the true prevalence of covert PUR is unknown [4–6] and possibly the reason that little is known about its natural course and potential long-term adverse effects.

The development of PUR is likely to be multifactorial; anatomical changes caused by delivery, such as bladder descent through pushing and pain due to birth-related pelvic floor trauma may disturb normal voiding by causing obstruction, loss of awareness of bladder filling, and inhibition of micturition. The possibility of the influence of these factors in the observed impairment of voiding might be reflected by the observation that the occurrence of PUR is higher in patients with epidural analgesia, episiotomy, and higher birth weight [6, 7].

Based on limited data about its transient natural course, some have hypothesised that covert PUR is a physiological phenomenon related to delivery [1, 8, 9]. Indeed, several authors have shown that elevated PVRVs generally normalise within days [1, 6, 9]. Still, even a single episode of bladder overdistension can create prolonged micturition problems and, rarely, even kidney failure [10, 11]. Yet, no studies have examined the bladder function of these women with covert PUR in the long term.

The question arises in which respect the puerperal lower urinary tract differs functionally from non-pregnant women [4, 9]. Together with the fact that one out of three parous women suffers from urinary incontinence and micturition problems in the first year after delivery [12], it seems relevant to study not only the normalisation of PVRVs directly postpartum, but also the influence of covert PUR on bladder function and micturition symptoms in the longer term.

To study these potential long-term effects of incomplete bladder emptying postpartum and to evaluate if the current definition is sufficient to discriminate between physiology and pathological conditions, a prospective cohort study was performed in women with and without covert PUR throughout the first year after their vaginal delivery.

## Materials and methods

A prospective observational study was performed between August 2011 and June 2013 in six teaching hospitals in the Netherlands. The study was approved by the medical ethics committee of the Academic Medical Centre in Amsterdam, the Netherlands (MEC AMC 10/277) and all participating centres. The study was registered in the Dutch Trial Registry (NTR 3118).

During the study period, the PVRV of women who had delivered vaginally was measured routinely after the first spontaneous void with a portable non-invasive transabdominal bladder ultrasound device (BVI 9400 Bladderscan®, Verathon Medical Europe, Ijsselstein, the Netherlands). When patients were able to void on a toilet, the quantity of the first micturition was measured. If women voided while showering, no measurement of the quantity of the first void could be obtained. Written informed consent was obtained from all patients. Patients with and without a PVRV ≥150 mL were included. Women with a PVRV >150 mL were included in the study group whereas women with a PVRV <150 mL acted as controls.

Initially, women were asked to participate after PVRV was measured. Although most of the women declined participation in this way, later on, the protocol was changed to the possibility of informing, and informed consent was obtained from patients before delivery.Patients aged <18 years, patients with insufficient knowledge of the Dutch language and patients with pre-existing kidney or bladder problems were excluded from the study.

### Measurements

All women with PVRV ≥150 mL were re-evaluated on day 4 after delivery (the day of delivery was set at day 0) and measured repeatedly until a residual volume of <150 mL was reached. If PVRV was still more than 150 mL on day 4, measurements were repeated at day 7 and weekly afterwards.

**Fig. 1:**
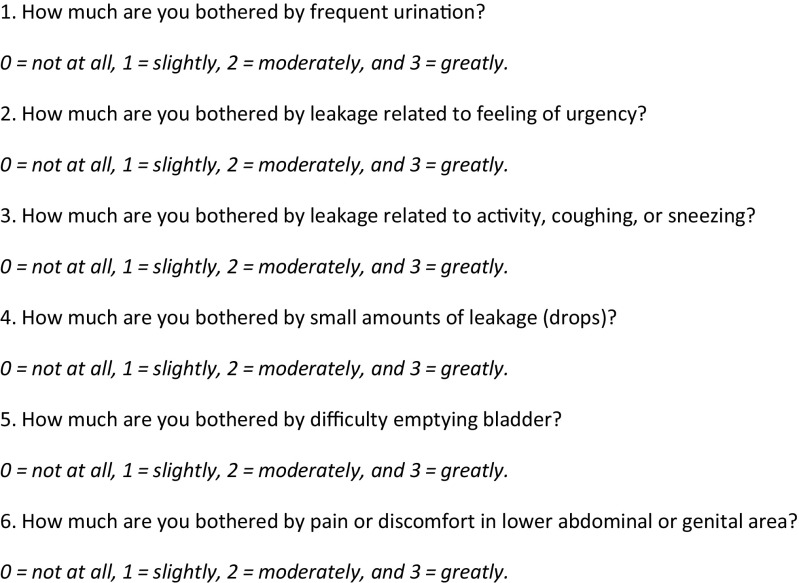
Urogenital Distress Inventory (UDI-6) questionnaire

To assess the presence of micturition symptoms, all patients were asked to complete the Dutch validated short version of the Urogenital Distress Inventory (UDI-6) on the day of delivery as well as on the 4th, 7th, 30th, 90th days and 1 year after delivery [13, 14].The UDI-6 is a six-item validated questionnaire used to identify symptoms associated with lower urinary tract dysfunction. The UDI questionnaires have previously been translated and validated in Dutch and are broadly accepted by urogynaecological clinicians in the Netherlands [14, 15]. Questionnaires were analysed using a four-point scale, which resulted in higher scores indicating more severe symptoms (Fig. 1) [16].

The UDI-6 can be divided into three domains: the first domain (questions 1 and 2) measures irritative symptoms such as frequency and urgency, the second domain (questions 3 and 4) measures incontinence symptoms and the third domain (questions 5 and 6) measures obstructive symptoms.

### Data and statistical analysis

It was hypothesised that women diagnosed with covert PUR would have micturition problems more frequently 1 year after delivery than women with a PVRV <150 mL.

A difference of 8 points in the total score of the UDI-6 was considered to be clinically relevant [17]. To achieve a power of 90% for detecting a significant difference with an α of 0.05 using a two-sided unpaired* t* test a total of 86 patients with asymptomatic urinary retention was needed for evaluation. During the study period, the drop-out rate was higher than anticipated. Therefore, the total number of patients was raised to 120 in each group.

Patient characteristics and the prevalence of micturition symptoms were compared between patients with and without covert PUR using an unpaired Student’s* t* test for continuous variables, a Mann–Whitney* U* test in the case of non-parametric distribution or a Pearson’s Chi-squared test for dichotomous variables (SPSS, IBM, version 23). We compared total mean scores of the total UDI-6 scores between the two groups. Univariate and multivariate logistic regression were used to identify confounding factors. Statistical significance was determined using Chi-squared testing. A *p* value of <0.05 was used as the statistical significance level.

To evaluate the contemporary definition of covert PUR (i.e. PVRV ≥150 mL after spontaneous micturition), as defined by Yip et al. in 1997 [4], the 75th and 95th percentiles of the measured PVRVs in women with PVRV ≥150 mL was calculated to assess the current classification.

We also evaluated median UDI-6 total and domain scores for PVRV ≥250 mL and PVRV ≥500 mL. These cut-off values were based on a previous study in which the median PVRV of an unselected cohort of 745 patients was 140 mL and the 75th and 95th percentile were 250 mL and 540 mL respectively [18]. As the currently used cut-off value, i.e. PVRV ≥150 mL, may be too low to discriminate between a pathological condition and physiology, we believed that adding these values would be clinically relevant.

## Results

A total of 241 patients were included: 122 patients with a PVRV ≥150 mL and 119 patients with PVRV <150 mL. Of these patients, data of 119 and 105 patients respectively could be used in the final analysis, owing to incomplete data.

Table 1 shows the baseline characteristics of the patients included. A PVRV ≥150 mL was more common among primiparous women than among multiparous women. Univariate regression analysis showed that only “any analgesia” was a potential confounder with an OR 1.87 (95% CI 1.116–3.138). All other clinical factors were not statistically significant. In the multivariate regression analysis, no independent confounding factors remained.

**Table 1:** Patient characteristics

Patient characteristics	PVRV <150 mL	PVRV ≥150 mL	*p* value
Nulliparous (%)	43	58	0.02
Gestational age^a^	39.3	39.4	NS
Maternal age (years)^a^	32	31.6	NS
Time of first stage (h)^b^	5	6	NS
Time of second stage (min)^b^	6	20	NS
Mean volume of first void (mL)^b^	370	350	NS
Spontaneous vaginal delivery (%)	85	84	NS
Assisted vaginal delivery (%)	15	16	NS
Birth weight (g)^a^	3,373	3,237	NS
Any analgesia (%)	35	41	NS
Epidural analgesia (% of total patients)	16	22	NS
Episiotomy (%)	18	16	NS

*PVRV* post-void residual volume, *NS* non-significant

^a^Mean

^b^Median

The median voided volume was 370 mL (range 100–1,100 mL) in women with PVRV <150 mL and 350 mL (range 50–1,900 mL) in women with PVRV ≥150 mL and did not differ significantly. The median PVRV was 175 mL in the total group and 300 mL in the group of women with a PVRV ≥150 mL. The 75th percentile of PVRV in the total study group was 303 mL and the 95th percentile was 549 mL. In patients with PVRV ≥150 mL, a total of 83 women had PVRV ≥250 mL and 19 women had PVRV ≥500 mL. This represents 68% and 16% of women respectively in the study group of women with PVRV ≥150 mL.

Women with PVRV ≥150 mL were re-evaluated on day 4 after delivery. On day 4, 92% of these women were able to completely empty their bladder. The PVRV of 8 out of a total of 100 women who returned at this visit remained above 150 mL (160–480 mL). Of these 8 women, 7 were able to void completely on day 7 and the final patient had a PVRV <150 mL at day 10 postpartum.

Table 2 lists the results of the UDI scores compared with women with PVRV ≥150 mL, presented as total scores and domain scores. For women with PVRV ≥150 mL, no statistical significant differences were found 1 year after delivery. Regarding domain scores, we found that the first questionnaire (day 0, i.e. day of delivery), women with PVRV ≥150 mL experienced more irritative symptoms (*p* < 0.001). For other differences in domain scores, no statistically significant difference was reached.

**Table 2:** Urogenital Distress Inventory (UDI-6) scores: women with PVRV <150 mL compared with women with PVRV ≥150 mL

	Day 0			Day 4			Day 7			Day 30			Day 90			Day 365		
	< 150 mL	≥ 150 mL	*p* value	< 150 mL	≥ 150 mL	*p* value	< 150 mL	≥ 150 mL	*p* value	< 150 mL	≥ 150 mL	*p* value	< 150 mL	≥ 150 mL	*p* value	< 150 mL	≥ 150 mL	*p* value
	*n* = 94	*n* = 119		*n* = 72	*n* = 100		*n* = 69	*n* = 77		*n* = 63	*n* = 68		*n* = 57	*n* = 64		*n* = 63	*n* = 80	
UDI-6 total score^a^	10.8 ± 14.5	11.4 ± 12.6	0.4	9 ± 11.2	8.6 ± 10.2	0.6	7.5 ± 7.6	9.4 ± 13.6	0.9	6.6 ± 9.2	6.1 ± 9.5	0.6	5.8 ± 8.7	8.0 ± 10.6	0.2	8.1 ± 11.1	9.8 ± 11.3	0.2
Irritative symptoms^a^	9.6 ± 18.7	13.6 ± 17.4	0.015	5.8 ± 11.2	7.5 ± 13.9	0.6	5.6 ± 10.9	7.6 ± 17.2	0.9	6.1 ± 11.8	3.9 ± 9.6	0.2	3.5 ± 8.2	6.3 ± 12.0	0.3	7.7 ± 14.3	10.4 ± 17.5	0.2
Stress symptoms^a^	11.5 ± 18	9.0 ± 15.6	0.2	7.2 ± 14.5	5.5 ± 10.9	0.8	12.6 ± 15.8	8.0 ± 17.0	0.6	7.4 ± 12.9	7.1 ± 13.6	0.7	8.8 ± 14.8	10.1 ± 16.4	0.6	10.6 ± 16.7	10.6 ± 17.0	0.8
Obstructive symptoms^a^	11.5 ± 20	11.8 ± 17.5	0.4	13.9 ± 18.5	12.7 ± 17.4	0.7	6.6 ± 9.2	12.6 ± 16.3	0.8	6.4 ± 11.4	7.1 ± 12.9	0.8	5.0 ± 9.9	7.6 ± 11.8	0.2	6.1 ± 12.8	8.5 ± 15.2	0.2

^a^Mean ± SD)

Results of the UDI scores for PVRV ≥250 mL and PVRV ≥500 mL are presented in Table 3; in addition to more stress symptoms in women with PVRV <250 mL, no statistical significant differences were identified.

**Table 3:** The UDI scores for PVRV ≥250 mL and PVRV ≥500 mL

	Day 0						Day 4						Day 7						Day 30						Day 90						Day 365					
	< 250 mL	≥ 250 mL	*p* value	< 500 mL	≥ 500 mL	*p* value	< 250 mL	≥ 250 mL	*p* value	< 500 mL	≥ 500 mL	*p* value	< 250 mL	≥ 250 mL	*p* value	< 500 mL	≥ 500 mL	*p* value	< 250 mL	≥ 250 mL	*p* value	< 500 mL	≥ 500 mL	*p* value	< 250 mL	≥ 250 mL	*p* value	< 500 mL	≥ 500 mL	*p* value	< 250 mL	≥ 250 mL	*p* value	< 500 mL	≥ 500 mL	*p* value
	*n* = 135	*n* = 78		*n* = 194	*n* = 19		*n* = 107	*n* = 65		*n* = 157	*n* = 15		*n* = 96	*n* = 50		*n* = 131	*n* = 15		*n* = 85	*n* = 46		*n* = 117	*n* = 14		*n* = 77	*n* = 44		*n* = 111	*n* = 10		*n* = 92	*n* = 51		*n* = 132	*n* = 11	
UDI-6 total score^a^	11.7 ± 14.2	10.2 ± 11.9	0.6	11.3 ± 13.6	9.4 ± 11.7	0.6	9.3 ± 9.8	7.8 ± 10.0	0.2	8.5 ± 9.3	11.5 ± 14.8	0.7	7.6 ± 8.1	10.1 ± 15.4	0.9	7.5 ± 8.3	17.0 ± 23.7	0.3	6.6 ± 8.8	5.8 ± 10.3	0.3	6.2 ± 8.6	7.14 ± 15.6	0.6	7.2 ± 10.8	6.4 ± 7.8	0.7	6.9 ± 9.9	7.2 ± 8.7	0.6	9.4 ± 12.1	8.5 ± 9.6	0.9	9.3 ± 11.4	7.1 ± 8.9	0.7
Irritative symptoms^a^	11.4 ± 18.9	12.6 ± 16.4	0.2	11.9 ± 18.3	10.5 ± 14.9	0.9	7.3 ± 12.9	5.9 ± 12.6	0.4	6.5 ± 12.2	10.0 ± 18.7	0.5	4.9 ± 9.3	8.3 ± 19.1	0.7	4.6 ± 8.8	18.9 ± 31.4	0.2	5.5 ± 11	4.0 ± 10.1	0.3	4.7 ± 9.9	9.5 ± 19.3	0.9	4.8 ± 10.8	5.3 ± 10.0	0.5	4.8 ± 10.4	6.7 ± 11.7	0.5	9.6 ± 16.9	8.5 ± 14.7	0.9	9.5 ± 16.7	6.1 ± 8.4	0.9
Stress symptoms^a^	12.1 ± 17.6	6.6 ± 14.5	0.008	10.6 ± 17	4.3 ± 12.2	0.07	7.5 ± 14.2	4.0 ± 8.8	0.2	6.4 ± 12.7	4.4 ± 9.9	0.6	5.9 ± 11.6	8.7 ± 18.8	0.7	6.1 ± 11.9	13.3 ± 28.3	0.6	7.3 ± 12.7	7.3 ± 14.4	0.7	7.0 ± 12.4	4.8 ± 13.8	0.9	10.4 ± 17.5	8.0 ± 11.6	0.9	9.6 ± 16	8.3 ± 11.8	0.9	12 ± 18.5	8.2 ± 13.1	0.3	10.7 ± 17.1	9.1 ± 13.7	0.9
Obstructive symptoms^a^	11.9 ± 18.8	11.3 ± 18.3	0.8	11.5 ± 18.4	13.1 ± 21.1	0.7	13.1 ± 17.3	13.3 ± 18.9	0.8	12.5 ± 16.8	20.0 ± 26.1	0.3	12.1 ± 15.2	13.3 ± 17.5	0.9	11.8 ± 15.3	18.9 ± 20.8	0.2	7.1 ± 12.1	6.2 ± 12.4	0.6	7.0 ± 12.0	7.2 ± 8.7	0.2	6.5 ± 11.5	6.1 ± 10.2	0.9	6.3 ± 10.8	6.7 ± 14.1	0.7	6.7 ± 14.6	8.8 ± 13.5	0.1	7.6 ± 14.2	6.1 ± 15.4	0.5

^a^Mean ± SD

## Discussion

This study shows that many women experience inadequate voiding directly after vaginal delivery. PVRVs up to 1,000 mL have been measured in postpartum women after spontaneous micturition. 92% of women with PVRV >150 mL after the first void were able to adequately empty their bladder on the 4th day after vaginal delivery. Regarding long-term micturition problems, 1 year after vaginal delivery, no statistical significant differences could be found in women with PVRV ≥150 mL compared with women with PVRV <150 mL.

Our study supports the findings in previous publications that covert PUR is a common phenomenon after vaginal delivery. Moreover, this study confirms that abnormal PVRVs after vaginal delivery “recover” spontaneously after several days, as 92% of our population were able to empty their bladder sufficiently 4 days after delivery [1, 6, 9, 19, 20]. In patients with prolonged PUR (i.e. persistently increased PVRVs after 4 days, *n* = 8), we found no statistical significant differences in UDI total or domain scores or patient characteristics compared with the control group.

In the group with PVRV ≥150 mL, 15% of women had a PVRV ranging from 150 to 250 mL, 69% (*n* = 86) had a PVRV between 250 and 500 mL and 16% (*n* = 19) of women had a PVRV above 500 mL. This implies that in clinical daily practice, a considerable percentage of women with high residual volumes remain unnoticed.

With a median PVRV of 175 mL after the first void in the total group and median PVRV of 300 mL in the group of women with a PVRV ≥150 mL, it is reasonable to assume that the cut-off value of 150 mL, as used by many authors [1, 4] and based on residual volumes in non-pregnant patients, may be too low to detect a pathological condition. Many authors focus on abnormal PVRVs; however, an internationally accepted definition with clinically significant threshold values is lacking [21]. Although large PVRVs (≥ 200–300 mL) may suggest a higher incidence of lower urinary tract symptoms (LUTS), evidence for using this measurement as a predictor is low [22]. It seems reasonable to define adequate emptying of the bladder as a voided volume that is over two thirds of the bladder capacity, indicating that residual volumes over 30% of the bladder volume could be abnormal. With non-pregnant women with bladder capacities around 400–500 mL, this would result in PVRV <150 mL. However, as bladder volumes during pregnancy and postpartum can reach and exceed 1,000 mL, it is a reasonable assumption that limiting PVRVs to 150 mL after delivery is clinically irrelevant. As over 15% of our patients had PVRV ≥500 mL, comparable with previous studies [18, 19], we believe that the cutoff value to detect a potential pathological condition should be increased. We therefore suggest that the new definition of covert PUR should be: “covert or asymptomatic postpartum urinary retention (PUR) includes post void residual volume (PVRV) ≥ 500 mL after the first spontaneous void following (vaginal) delivery, measured by ultrasound or catheter”. This adjusted definition could help us to learn more about potential LUTS related to inadequate bladder emptying after (vaginal) delivery and should therefore not only be a topic for future research, but also be widely accepted by international expert societies and incorporated into daily clinical guidelines.

To our knowledge, our study is the first to use a validated questionnaire to examine LUTS in women with increased PVRV after the first spontaneous micturition following vaginal delivery [16]. Previous studies have mainly focused on the prevalence and transient course of covert PUR [1, 4, 6]. As far as we know, only Yip and colleagues published results on their evaluation of long-term micturition problems in women with postpartum urinary retention, 4 years after childbirth [23]. In this study, no significant differences in the occurrence of urinary incontinence were detected. However, a few remarks can be made. Besides the use of non-validated questionnaires, no distinction was made between women with covert and those with overt PUR. Although both phenomena refer to inadequate voiding after delivery, the pathophysiology of the inability to void (overt PUR) and incomplete bladder emptying (covert PUR) may be different, resulting in different management strategies and potential long-term consequences.

The results of our study are also consistent with published studies on bothersome LUTS in women 1 year after delivery [12, 24]. In our total population, 42% of women experienced some bladder symptoms, with 12% having bothersome irritative symptoms, 15% moderate to severe stress-related complaints and 11% moderate to severe symptoms of obstruction or discomfort. Although we have no information on the prevalence of LUTS before or during gestation in our patients, we believe that our study population is a reliable representation of the general population.

Although the pathophysiology of urinary retention is still unknown and probably multifactorial, we can learn from our results. With a median first voided volume of 355 mL (± 263 mL) and a median PVRV of 174 mL (± 176 mL), women in our study showed a much higher bladder capacity than those found in non-pregnant females [22, 25]. Although most of our patients showed large voided volumes, up to 1,900 mL, we think that it is reasonable that these findings reflect the physiology of pregnancy, as pregnancy is characterised by drastic changes to several organs and organ systems. Not only are there anatomical changes such as dilation of the ureters and calyces of the kidneys, but also several functional changes such as increased glomerular filtration and urinary output. The bladder capacity most likely adapts to these new functional requirements [26]; being attached to the growing uterus, it has to adapt to this stretch and extra urine production to prevent overdistension and facilitate extra storage function. The latter possibly prevents bothersome frequency so that pregnant women are able to maintain relatively normal daily activities.

Another factor may be the influence of hormonal changes. Progesterone and relaxin may be (partially) accountable. Relaxin is a peptide hormone of the insulin-like growth factor family and has been associated with collagen remodelling and adjustments of renal physiology and vasodilatation in normal pregnancy. In the 48 h before labour, relaxin causes rapid depolymerisation of collagen bonds to the point where the collagen loses 95% of its strength, allowing the vagina to stretch and its supporting structures to expand sufficiently for vaginal delivery [27]. It is not unthinkable that relaxin has an effect on bladder characteristics too. Progesterone is a more well-known gestational hormone that is not only important in retaining pregnancy, but also reduces urethral tone, peristalsis and contraction pressure of the bladder, potentially resulting in enlargement of bladder capacity in the puerperium [28].

Some limitations of our study need to be discussed, first of all the large number of incomplete questionnaires. Despite repeated invitations by (e-)mail or phone, numerous patients did not react to these reminders to respond. Initially, our sample size calculation was based on 15% dropping out. However, during the study period we experienced that up to 35% of the patients included were lost to follow up. Therefore, it was necessary to file an amendment to reach the required number of patients. Although we eventually were not able to collect the initially required 86 complete questionnaires per group 1 year after delivery, resulting in insufficient power of our data, we believe that our results are reliable. We found no statistical significant differences between the patients who were lost to follow-up and the patients who completed all questionnaires. Our sample size calculation was based on finding a difference of 8 points in the total UDI score. However, with a mean UDI total score of 8.1 ± 11.1 in women with PVRV <150 mL and mean 9.8 ± 11.3 in patients with PVRV ≥150 mL, a difference of 8 points does not seem to be clinically relevant in this population, as most of the women did not experience LUTS, consistent with previous studies [12, 29]. It is possible that the group of women with complaints were more inclined to respond, generating an information bias. However, the possible effect of this bias is considered to be small, as the distribution of incomplete data was equal between the group with and without abnormal PVRV. We therefore believe that although our results may be statistically underpowered, we have shown that PVRV ≥150 mL is not related to increased urinary-related problems 1 year after delivery.

A second limitation could be the time of measurement of the PVRV. According to our study protocol this was performed after the first spontaneous void after delivery. In other studies this was done after 24 h and up to 72 h after delivery, resulting in lower prevalences of covert PUR [4, 19, 20]. Therefore, it is possible that our results might have overestimated the occurrence of abnormal PVRV. However, as most women who deliver vaginally leave the hospital within a few hours and the ability to void spontaneously is used as an indication for adequate bladder function, we believe that if the PVRV is measured, it is only feasible to do so after the first spontaneous micturition.

Another limitation could be the potential selection we have created by only including patients delivering in the hospital. This could possibly mean that we selected women who have had more complicated deliveries than women without a medical history or medical indication to deliver in the hospital.

Last, we were not informed of our patient’s individual “normal” PVRV and “normal” UDI-6 scores before delivery and pregnancy. However, it seems unlikely that these otherwise healthy young women would have had urinary retention-related symptoms in their earlier life. We therefore believe that this does not influence our results to a great extent.

The results of our study confirm previous studies that covert PUR in women after vaginal delivery normalises spontaneously within several days [1, 4, 6, 20] and that women with PVRV ≥150 mL do not experience more LUTS 1 year after delivery.

Although we believe that the cut-off value of PVRV ≥150 mL is insufficient to distinguish between physiology and pathology and is based on limited data in non-pregnant patients, we suggest adjusting the definition of covert PUR and incorporating it into daily clinical practice. Women at risk of covert PUR, defined as PVRV ≥500 mL after the first spontaneous void after (vaginal) delivery, should be the focus of future research to learn whether these patients could benefit from screening and treatment [6, 18].

## Conclusions

Covert PUR according to the definition of PVRV ≥150 mL, is a common and transient phenomenon after vaginal delivery that does not result in more LUTS 1 year after delivery. Although the current definition is not useful in identifying postpartum those women who have a pathological condition, we suggest changing the definition of covert PUR to: “post-void residual volume ≥ 500 mL after the first spontaneous void after (vaginal) delivery”. This cut-off value is the value at which some women do need more time to normalise emptying of the bladder. The exact clinical implications of covert PUR need to be further studied in this subcategory of women.
